# MRI Screening in Vestibular Schwannoma: A Deep Learning-based Analysis of Clinical and Audiometric Data

**DOI:** 10.1097/ONO.0000000000000028

**Published:** 2023-03-09

**Authors:** Sarah Kortebein, Shoujun Gu, Kathy Dai, Elizabeth Zhao, Kristal Riska, David Kaylie, Michael Hoa

**Affiliations:** 1 Department of Head and Neck Surgery and Communication Sciences, Duke University School of Medicine, Durham, NC; 2 Auditory Development and Restoration Program, NIDCD Otolaryngology Surgeon-Scientist Program, Division of Intramural Research, NIDCD/NIH, Bethesda, MD.

**Keywords:** Machine learning, Skull base, Vestibular schwannoma

## Abstract

**Objective:**

To find a more objective method of assessing which patients should be screened for a vestibular schwannoma (VS) with magnetic resonance imaging (MRI) using a deep-learning algorithm to assess clinical and audiometric data.

**Materials and Methods:**

Clinical and audiometric data were collected for 592 patients who received an audiogram between January 2015 and 2020 at Duke University Health Center with and without VS confirmed by MRI. These data were analyzed using a deep learning-based analysis to determine if the need for MRI screening could be assessed more objectively with adequate sensitivity and specificity.

**Results:**

Patients with VS showed slightly elevated, but not statistically significant, mean thresholds compared to those without. Tinnitus, gradual hearing loss, and aural fullness were more common in patients with VS. Of these, only the presence of tinnitus was statistically significant. Several machine learning algorithms were used to incorporate and model the collected clinical and audiometric data, but none were able to distinguish ears with and without confirmed VS. When tumor size was taken into account the analysis was still unable to distinguish a difference.

**Conclusions:**

Using audiometric and clinical data, deep learning-based analyses failed to produce an adequately sensitive and specific model for the detection of patients with VS. This suggests that a specific pattern of audiometric asymmetry and clinical symptoms may not necessarily be predictive of the presence/absence of VS to a level that clinicians would be comfortable forgoing an MRI.

Acoustic neuromas or vestibular schwannomas (VSs) are benign neoplasms that originate from the Schwann cells enveloping the vestibular component of the eighth cranial nerve. These tumors commonly present in the fourth–sixth decades of life with an equal distribution between males and females and a prevalence of 1–5/100,000 people (1–3). There are sporadic and a genetic forms. The genetic form is found in patients with Neurofibromatosis type 2 (NF2) and is usually bilateral. These tumors can present with asymmetrical sensorineural hearing loss (ASNHL), unilateral tinnitus, aural fullness, vestibular symptoms, and facial paresthesias. Contrast enhanced magnetic resonance imaging (MRI) is the gold standard for imaging and diagnosis of VS with a sensitivity and specificity nearing 100% (4). There is no universal definition of asymmetric SNHL or well described method with high specificity and sensitivity for determining which patients should receive an MRI (5,6). Positive imaging rates as low as 1.5% have been reported (5,7). The cost of an MRI brain with contrast varies between $500 and 8000 depending on insurance coverage and location (8). T2 weighted MRI’s without contrast have been proposed as a more cost effective screening tool; however, the rate of negative imaging does not change (9).

Prior studies have examined various clinical and audiometric parameters associated with higher rates of VS detection on MRI (Table 1) (2,10–19). Saliba et al proposed a “rule 3000,” which suggested imaging patients with an asymmetry of ≥15 dB at 3 kHz, detecting VS in about 2% of MRI scans (2). Pena et al compared a positive and negative cohort of Veterans Affairs patients matched in age, sex, combat experience, and comorbidities and found a >80% specificity for VS when imaging was done for asymmetry of ≥45 dB at 3 kHz, asymmetry of ≥15 dB at 3 kHz with unilateral; or 80% or more asymmetric word recognition, but the sensitivity using this criteria was 27%–43% (20).

**TABLE 1.** Definitions of asymmetric SNHL reported in the literature

Asymmetry of thresholds	Name	References
Single-frequency comparisons		
≥20 dB at any single frequency between 0.5 and 4 kHz	UK Department of Health	Nouraei et al (10)
≥15 dB at any single frequency between 0.5 and 4 kHz	Nashville	Welling et al (12)
≥15 dB at any single frequency	AMCLASS-B-Urben	Margolis and Saly (13); Urben et al (14)
≥15 dB at 3 kHz	Rule 3000	Saliba et al (2,6)
≥15 dB at 4 kHz	Rule 4000	
Two adjacent frequency comparisons		
≥20 dB at any 2 neighboring frequencies	Sunderland	Dawes and Jeannon (16)
≥15 dB at 2 or more frequencies, OR ≥ 15% difference between speech discrimination	Cueva	Cueva (17)
≥10 dB at 2 or more frequencies	AMCLASS-A-Urben	Margolis and Saly (13); Urben et al (14)
Averaged multiple frequency comparisons		
≥15 dB between ears averaging 0.5 to 3 kHz	AAO-HNS	AAO-HNS
≥15 dB between ears averaging 0.5 to 8 kHz	Oxford	Sheppard et al (18)
≥15 dB between ears averaging 1 to 8 kHz	Seattle	Hunter et al (19)

AAO-HNS indicates American Academy of Otolaryngology Head and Neck Surgery; AMCLASS, audiogram classification system; OR, odds ratio; SNHL, sensorineural hearing loss.

In this study, we approached the longstanding challenge of identifying which patients require MRI screening for VS using a novel machine learning method to predict the presence of a VS based on audiometric and clinical variables. Machine learning or deep learning-based algorithms are a form of artificial intelligence that develop automated computer algorithms to predict outcomes. Performance of these algorithms is graded on their level of discrimination, or probability of predicting the correct outcome, and their calibration, or the degree of over or under estimation of the predicted versus true outcome (10).

## MATERIALS AND METHODS

### Data Collection

The protocol for this study was submitted to the Duke University Institutional Review Board and approved. Information regarding each patient’s presenting symptoms including unilateral or bilateral non pulsatile tinnitus, gradual (GHL), or sudden hearing loss (SHL), aural fullness dizziness; and past medical history including noise exposure, history of surgery or radiation exposure to the inner ear, trauma to the middle/inner ear and diagnoses such as Meniere disease was collected by chart review. The presence of a VS was confirmed on MRI. Patients were included in the study if they were over the age of 18, had a complete hearing evaluation including air and bone conduction pure tone testing, speech reception threshold and word recognition; and an MRI confirming or disproving the diagnosis of VS. Patients with NF2 or without pretreatment audiometry were excluded. Comparisons were made between the nontumor and tumor ears.

### Audiometric Ratios

**FIG. 1.**
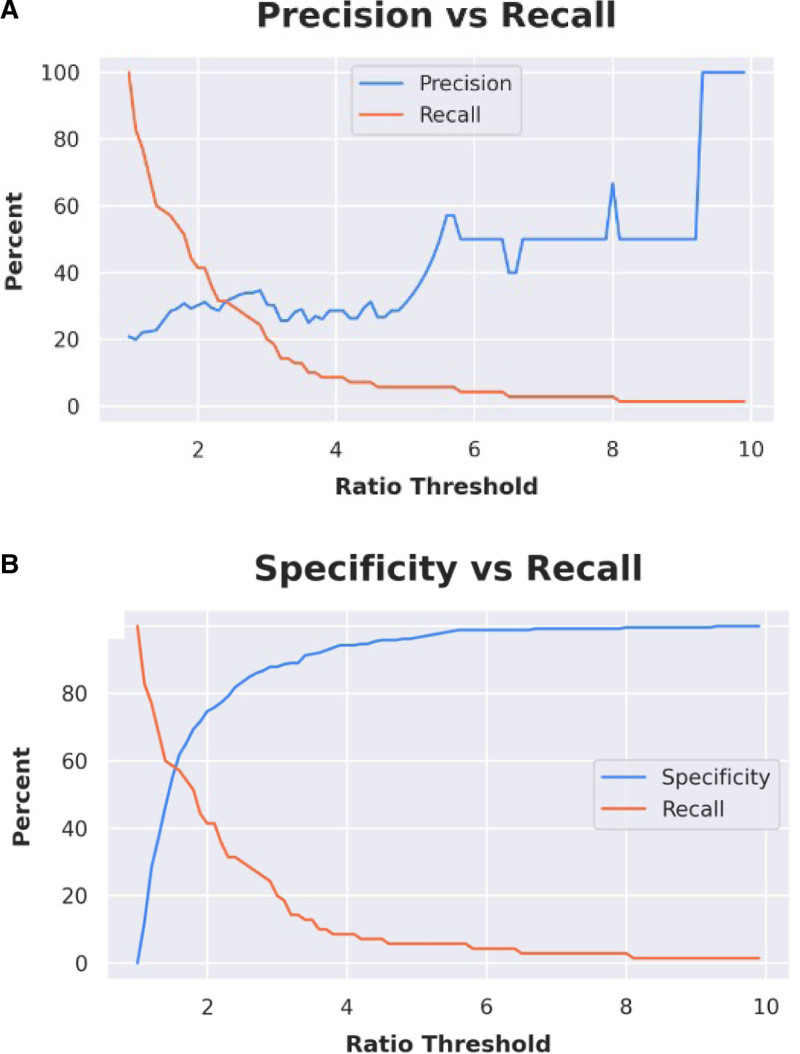
Assessment of high-frequency threshold average ratio as a method for detecting vestibular schwannoma.

An auditory ratio was created, defined as the average air conduction thresholds at 2000, 3000, 4000, 6000, and 8000 Hz in the ear with the tumors divided by the same average air conduction threshold in the ear without the tumor (Fig. 1). This was used to evaluate whether average auditory threshold differences in the mid to high frequencies between tumor and nontumor ears was predictive of which patients should be screened. Using the auditory ratios as potential predictive values for the presence or absence of tumors, we then plotted precision or positive predictive value (true positives over all patients identified as having a tumor by the given ratio threshold) curves depicted in blue and recall or sensitivity (true positives over all patients with tumors) curves depicted in orange in Figure 1.

### Data Processing

While many methods to account for missing data in machine learning- and deep learning-based analyses have been developed, the most straightforward is to start the analysis with the most complete dataset possible. Therefore, only variables with ≤ 5% missing data across the entire raw dataset and the following features were kept for analysis (both left and right): “wordrec,” “AC freq” (frequencies in Hz: “250,” “500,” “1000,” “2000,” “4000,” “8000”), “BC freq” (frequencies in Hz: “500,” “1000,” “2000,” “4000”), “tinnitus,” asymmetric hearing loss (AHL), “fullness,” SHL, and GHL.

The raw data set consisting of 1184 ears (592 patients) was then cleaned by filtering samples with error values. Specifically, the raw dataset was filtered in parallel by the following criteria: (1) ears with word recognition scores (wordrec) >100 or <0; and (2) ears with any values for the air conduction frequency threshold (AC freq) at 250, 500, 1000, 2000, 4000, or 8000 Hz >120 or <0 were removed. The result of these parallel filtering steps were merged to create a subset of the dataset without missing variable data. Reporting of symptoms (“tinnitus,” ahl, “fullness,” SHL, and GHL) was complete across all ears and filtering was not required. These filtering steps resulted in a final dataset consisting of 860 ears (430 patients).

### Machine Learning, Deep Learning and Accounting for Missing Data

A detailed description for these portions of the methods can be found in the Supplemental Methods Section, http://links.lww.com/ONO/A16.

### Data Visualization

All data visualizations including PCA were performed using Matplotlib (v3.5.1), Seaborn (v0.11.2), and Scikit-Learn (v1.0.2). PCA analysis (clustering display) was performed utilizing Scikit-Learn (v1.0.2) with default settings utilizing the features with ≤5% missing data as described above.

## RESULTS

### Demographics and Patient Subgroups

A total raw dataset of 1184 ears (592 patients) with audiograms completed within the Duke University Hospital system between January 2015 to January 2020 were included in the full chart review, including 158 ears with MRI-confirmed presence of VS. After filtering for complete symptom and audiometric data as detailed above, the filtered dataset consisting of 860 ears (430 patients), including 752 ears with MRI-confirmed absence of VS and 108 ears with MRI-confirmed presence of VS was analyzed using a deep-learning algorithm. Of the 108 tumors, 27 tumors were intracanalicular, 45 of these tumors were small (<1.5 cm), 25 tumors were medium (1.5–2.5 cm), and 11 tumors were large (>2.5 cm).

### Presenting Symptoms and Audiometric Data

**FIG. 2.**
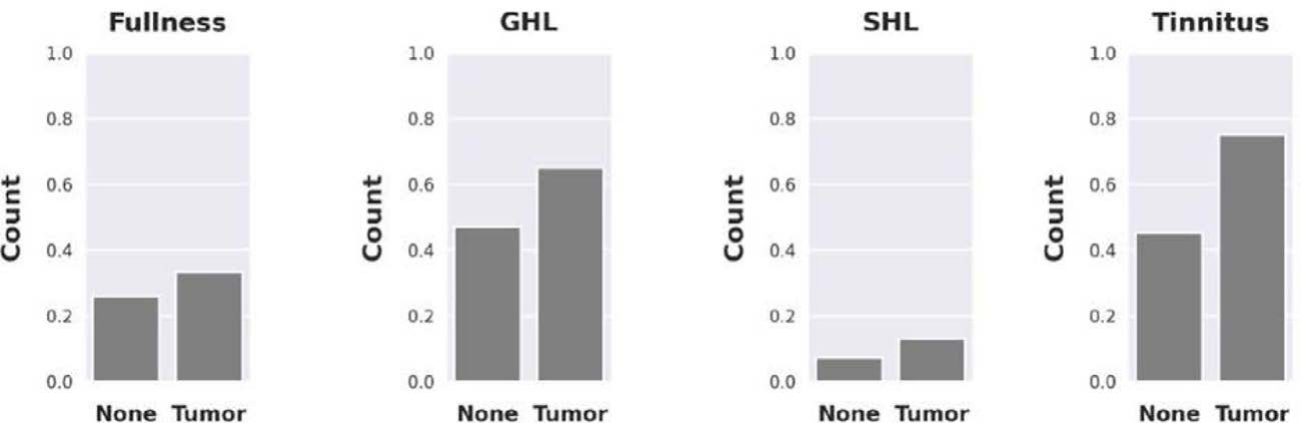
Differences in presenting symptoms in ears with and without a vestibular schwannoma including ear fullness, gradual hearing loss (GHL), sudden hearing loss (SHL), and tinnitus (*P* < 0.01).

**FIG. 3.**
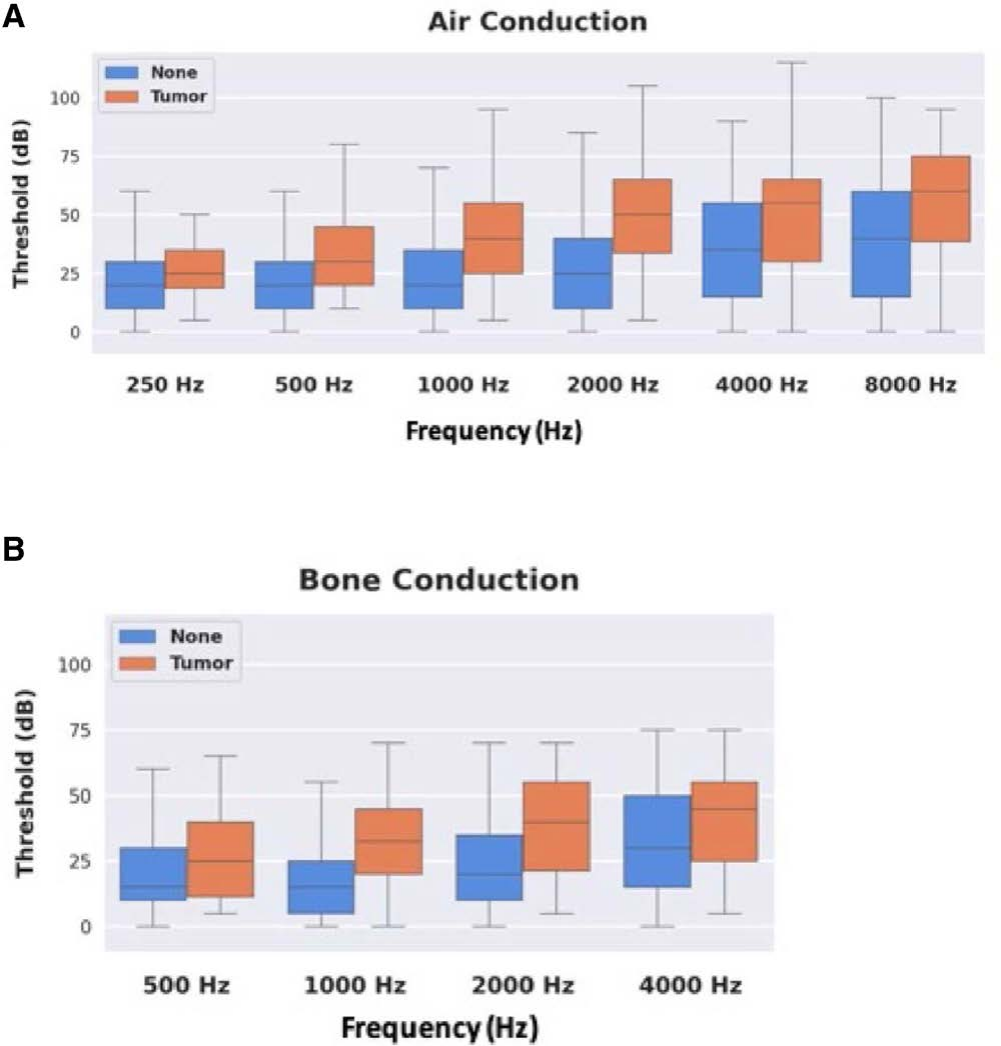
Air (*A*) and bone (*B*) conduction frequency threshold differences between ears with MRI-confirmed presence and absence of vestibular schwannoma (VS).

When comparing presenting symptoms tinnitus, aural fullness, and GHL showed the greatest differences between ears with and without a tumor (Fig. 2), but only tinnitus showed statistical significance (OR 1.659, *P* = 0.002). Unilateral and bilateral nonpulsatile tinnitus were grouped together for this evaluation. Dizziness was not localized by ear. There was no clear difference in occurrence of dizziness between tumor and nontumor patients. When comparing audiometric thresholds of ears with and without tumors, both groups had similar ranges for air and bone conduction. Ears with tumors did show slightly elevated mean thresholds, but this difference did not reach statistical significance (Fig. 3).

### PTA and Audiometric Ratios

As seen in Figure 3, comparison of average air conduction thresholds in the mid to high frequencies (1000–8000 Hz) for tumor versus nontumor ears has minimal utility. The precision curves in blue suggest that as the ratio asymmetry rises, the positive predictive value for the prevalence of a VS also rises. However, correspondingly, the recall curves demonstrate that, as the ratio asymmetry rises, the sensitivity of this measure declines. This was seen when comparing low frequency and all frequencies as well (data not shown). To find a ratio with a positive predictive value for finding a tumor near 100%, the sensitivity became so low that most tumors were missed. Thus, there does not appear to be a ratio that could predict the presence of a tumor with sufficient accuracy to forgo imaging.

### Deep Learning Analysis

**FIG. 4.**
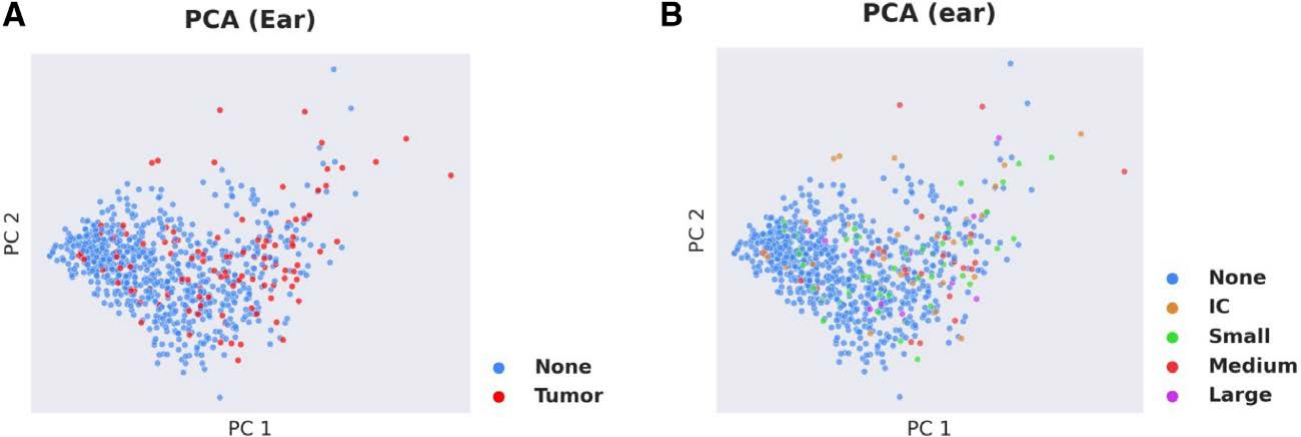
Deep-learning-based analysis of clinical and audiometric data in patients with and without vestibular schwannoma (VS) as confirmed by MRI.

**FIG. 5.**
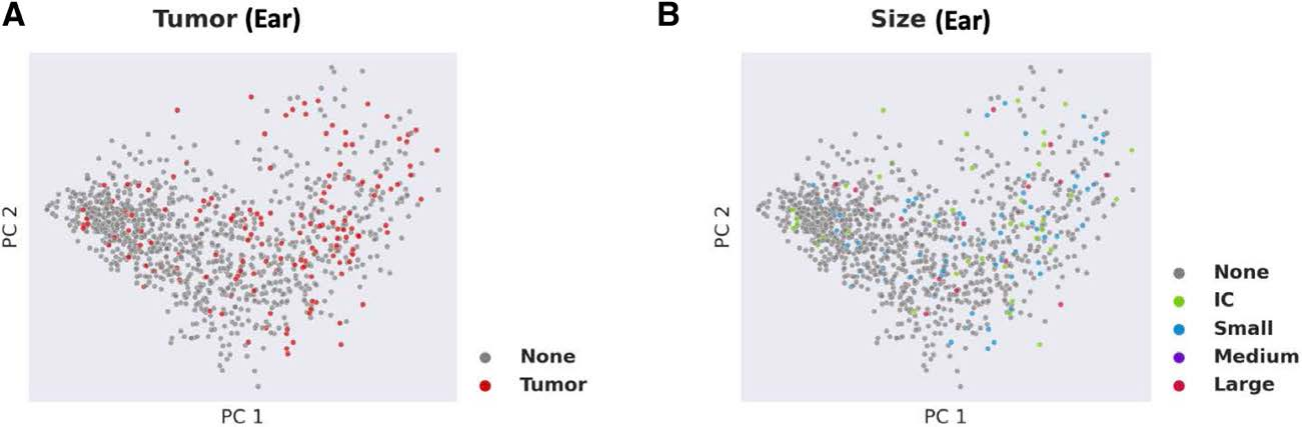
Deep-learning-based analysis of clinical and audiometric data in patients with and without vestibular schwannoma (VS) as confirmed by MRI using mean substitution for missing data in full patient cohort (592 patients) by ear.

The deep learning-based analysis, which utilized all variables in the filtered dataset, did not reliably distinguish between nontumor and tumor ears. As shown in Figure 4A, ears with tumors (red dots) did not cluster directly from ears without tumors (blue dots) on the basis of presenting symptoms and audiometric measures. The deep learning-based analysis was also not able to distinguish a difference between ears based on tumor size (Fig. 4B). Comparison of machine- and deep-learning-based approaches revealed improved accuracy and F1 scores, both measures for classification model performance, for the deep-learning-based approach (Supplemental Figure 1, http://links.lww.com/ONO/A16). To analyze the entire dataset, including the patients initially excluded due to missing values, we employed a mean substitution of missing data as a method for analyzing datasets with missing values as reviewed in Mirza et al (21). Our analysis of the total dataset using this methodology did not demonstrate distinct clustering of patients or ears with tumors from those patients or ears without tumors (Fig. 5).

## DISCUSSION

Detection of VSs is important to prevent complications that could result in further cranial nerve involvement or more severe consequences, such as, cerebrospinal outflow obstruction and herniation. However, thousands of patients per year receive an MRI to exclude a VS and only 1-5% are diagnosed on imaging. For this reason, it is important to have a sensitive screening tool to decipher which patients require a screening MRI. A more specific screening tool to select which patients require imaging screening would lower the high number of negative MRIs and reduce costs. Many attempts have been made in the literature to create a more specific clinical screening process based on symptoms and/or audiometric data, but none have proven to be both adequately sensitive and specific. This study used a deep learning-based analysis to assess whether any factors in a patient’s presenting clinical or audiometric data could be used to objectively determine whether a patient requires screening for a VS with adequate sensitivity and specificity. Deep learning-based analyses use multiple layers of algorithms to progressively transform raw data into more abstract representations of the data with the goal of finding features that helps disentangle the data for better, more accurate output. These analyses are inspired by biologic networks, such as the human brain, which uses layers of information to come to a conclusion or output. A training dataset is needed to first allow the deep learning-based model to find the connections that will produce the most accurate output. A test dataset is then used to test the algorithm. When an accurate deep-learning analysis is built, it is not possible to pinpoint which features of the raw data allowed the algorithm to make this accurate prediction, similar to how a human brain is able to recognize distinct faces—there are obvious differences, but the nuances are lost in the neural network of our brains.

In the current study, using a filtered dataset of 860 ears (430 patients), including 752 ears with MRI-confirmed absence of VS and 108 ears with MRI-confirmed presence of VS, a deep learning-based analysis was not able to differentiate between patients with and without a tumor based on their presenting audiometric data and symptoms. One hundred sixty-two patients were excluded due to incomplete audiometric data. Machine and deep learning analyses are only as good at the data utilized and incomplete data can skew these analyses. Several methods have been developed to account for missing data in machine learning approaches, the most straight forward of which would be to select the group of patients with the most complete data and exclude samples with missing data (21). We did this by examining the patient data variables that had the least missing data and then selecting the patients who had complete data with respect to those variables. Other acceptable methods to account for missing data in machine learning approaches include, but are not limited to, replacement with mean, median or mode values; hot-deck imputation; regression imputation; and K-nearest neighbor (21). Our analysis of the total dataset replacing the missing values with the mean values for each variable did not yield a different conclusion from our analysis of the dataset with complete data (Fig. 5). These analyses suggest that our approach with the subset of patients with the most complete dataset is valid.

### Audiometric Outcomes

Numerous audiometric protocols have been proposed as an objective approach to deciding which patients should receive a screening MRI for VS. A study by Cheng et al applied 15 of these proposed audiometric protocols to their cohort of 1751 cases with complete audiologic and radiologic data (11). These protocols classify patients based on differences at a single frequency, averages of several frequencies or at neighboring frequencies ranging from 10 to 30 dB (Table 1). None of the protocols achieved 100% sensitivity or specificity. Sensitivity rates ranged from 81% to 93% with a risk of false-negative cases ranging from 7% to 19%. Specificity rates ranged from 33% to 68% with a false-positive rate of 34% to 68%. These protocols trade off the choice between lowest clinical risk using a test with high sensitivity and high specificity. A recent machine learning study that used audiometric data alone did find similar sensitivity and specificity for commonly used rule-based evaluations to those found in the previous literature (22). A screening process with high sensitivity is important in the context of VSs because a missed tumor has severe consequences. If a tumor is found early, there are more choices for treatment including possible hearing preservation surgery or radiation treatment leading to better clinical outcomes for patients. If a tumor is allowed to progress it can lead to severe consequences, such as, hydrocephalus or herniation. Conversely, a screening process with high specificity avoids unnecessary, costly work up.

In our cohort, the differences between thresholds for ears with and without tumors did not reach significance. As the specificity of a screening model increases, the sensitivity falls, allowing patients with a VS to slip through the screening model (Fig. 3). The deep-learning-based assessment, which took into account all of the audiometric data for each ear including the pure tone thresholds at all frequencies, word recognition and speech threshold, could not separate the affected ears on a principal component analysis. This variable presentation is likely why accurately separating these patients based on objective data is difficult. Currently, there is no universally agreed upon method for selecting which patients should be screened for VS based on their audiometric outcomes. Clinicians are left to use their clinical judgment to weigh the risks and benefits of available screening tools.

### Clinical Presentation and Symptoms

Some proposed protocols for screening patients include clinical presenting symptoms like subjective hearing loss, tinnitus, aural fullness, or dizziness to further increase the diagnostic yield of MRI. A study by Ahsan et al performed a retrospective review of 451 patients with ASNHL who all underwent MRI screening in attempt to find clinical predictors of abnormal MRI findings (1). They found that reported vertigo/dizziness, tinnitus, and sudden SNHL were associated with abnormal MRI (*P* = 0.1–0.5). In the present study presenting clinical symptoms including subjectively reported ipsilateral or bilateral tinnitus, dizziness, asymmetrical hearing loss, unilateral or bilateral aural fullness, and unilateral or bilateral SHL or GHL were recorded for all patients. Patients with confirmed tumors were found to present with more symptoms overall and the symptoms that showed the greatest difference between tumor and nontumor ears were tinnitus, aural fullness, SHL, or GHL. The only rate of presentation that rose to statistical significance was tinnitus (Fig. 2). While these symptoms are subjective, they have historically been used in proposed screening algorithms for VSs, and, therefore, were used here in the deep-learning algorithm for completeness. When combining all clinical presenting symptoms with associated audiometric data in a deep-learning-based assessment the model was not able to differentiate patients with and without tumors with appropriate sensitivity and specificity. While clinical symptoms do accompany these tumors, and it seems physiologically sensible to use symptoms to aid in determining which patients require screening, there is large overlap between those symptoms in ears with and without tumors. The presence of these clinical symptoms do not increase the sensitivity and specificity of a clinical screening tool. This is likely because the presentation of these patients is variable, their symptoms overlap with patients that do not have a VS and a large number of patients are asymptomatic.

### Limitations

There are several limitations to this study. The clinical presentation and symptoms were all subjectively expressed by patients. The sensation of gradual or sudden hearing loss and dizziness were gathered from the chart review and not from serial audiograms or vestibular testing data. Serial audiogram data may be a future avenue for creating a better screening method for these tumors, but this study was meant to use the deep learning-based algorithm to determine if there was an objective way to predict the need for imaging based on presenting symptoms and initial audiogram. Another limitation could be that many of the nontumor audiograms were for patients presenting with hearing loss or otologic issues. A future study could include patients with normal hearing and no audiologic symptoms as a control. Adding vestibular data may also be something to consider in a future algorithm; however, most patients do not present with vestibular data, not all patients with asymmetrical hearing loss require vestibular testing, and the cost of this testing may not offset the cost of imaging. Adding more data points may be helpful in further distinguishing tumor and nontumor patients, however, in a deep-learning-based algorithm adding more data variables can make the model less predictive. It is best to have more subjects or training points than variables. When large datasets are available with abundant variables the algorithm will find a statistically significant result that is not logically, or in this case clinically, relevant. Another possible limitation is the number of patients and ears analyzed in this data set. We considered adding the data for more ears to the dataset; however, after analysis of the 430 patients/860 ears with complete data, it was evident that the deep learning-based algorithm was not able to differentiate between affected ears based with no identifiable separation between the groups on the principal component analysis. Furthermore, other machine learning-based classification methods demonstrated reduced performance with respect to accuracy and F1 scores, demonstrating reduced ability to accurately distinguish between affected ears. Adding a sizeable number of patients to the dataset, such as, 100,000 patients with a good proportion of them confirmed to have a VS may be able to change the capability of the algorithm, but VSs are not prevalent enough to gather this amount of data.

## CONCLUSIONS

Using audiometric and clinical data, deep learning-based analyses failed to produce an adequately sensitive and specific model for the detection of patients with VS. While a “golden ticket” for diagnosing a VS based on a patient’s clinical and audiometric data would be helpful, this study demonstrates that a better clinical screening model may not be possible using this data alone. However, biomarkers may be the future of diagnosis and treatment of these tumors instead of clinical presentation. Recent work has shown several potential biomarkers in the perilymph, actual tumors and CSF that show potential for use in diagnosis, but maybe more importantly prediction of potential for growth and even treatment of VS (23–26). Therefore, while audiometric and clinical data may not be able to further select for which patients require screening, there are other avenues that may be the future for screening and also treating VSs.

## FUNDING SOURCES

This research was supported (in part) by the Intramural Research Program of the NIH, NIDCD to MH (ZIA DC000088).

## CONFLICT OF INTEREST

MH is an Associate Editor for *Otology & Neurotology Open* and has been rescued from reviewing or making decisions for this manuscript.

## DATA AVAILABILITY STATEMENT

The datasets generated during and/or analyzed during the current study are not publicly available, but are available from the corresponding author on reasonable request.

## Supplementary Material



## References

[1] Ahsan SF Standring R Osborn DA Peterson E Seidman M Jain R. Clinical predictors of abnormal magnetic resonance imaging findings in patients with asymmetric sensorineural hearing loss. JAMA Otolaryngol Head Neck Surg. 2015;141:451–456. 25719460 10.1001/jamaoto.2015.142

[2] Saliba I Martineau G Chagnon M. Asymmetric hearing loss: rule 3,000 for screening vestibular schwannoma. Otol Neurotol. 2009;30:515–521. 19395982 10.1097/MAO.0b013e3181a5297a

[3] Marinelli JP Beeler CJ Carlson ML Caye-Thomasen P Spear SA. Global incidence of sporadic vestibular schwannoma: a systematic review. Otolaryngol Head Neck Surg. 2022;167:209–214. 34464224 10.1177/01945998211042006

[4] Dang L Tu NCY Chan EY. Current imaging tools for vestibular schwannoma. Curr Opin Otolaryngol Head Neck Surg. 2020;28:302–307. 32833884 10.1097/MOO.0000000000000647

[5] Gupta A Monsell EM. Which patients with asymmetric sensorineural hearing loss should undergo imaging?. Laryngoscope. 2018;128:1990–1991. 29392735 10.1002/lary.27118

[6] Saliba I Bergeron M Martineau G Chagnon M. Rule 3,000: a more reliable precursor to perceive vestibular schwannoma on MRI in screened asymmetric sensorineural hearing loss. Eur Arch Oto-Rhino-Laryngology. 2011;268:207–212. 10.1007/s00405-010-1378-9 20835831

[7] Wong BYW Capper R. Incidence of vestibular schwannoma and incidental findings on the magnetic resonance imaging and computed tomography scans of patients from a direct referral audiology clinic. J Laryngol Otol. 2012;126:658–662. 22578280 10.1017/S0022215112000680

[8] Vanvuren C. What Can Affect the Cost of an MRI?. Pensacola, FL: New Choice Health Inc.

[9] Ryan M Weissman JL Kaylie D. Is gadolinium contrast enhancement necessary in screening MRI for asymmetric sensorineural hearing loss? Laryngoscope. 2015;125:783–784. 25111873 10.1002/lary.24871

[10] Nouraei SAR Huys QJM Chatrath P Powles J Harcourt JP. Screening patients with sensorineural hearing loss for vestibular schwannoma using a Bayesian classifier. Clin Otolaryngol. 2007;32:248–254. 17651265 10.1111/j.1365-2273.2007.01460.x

[11] Committee on Hearing and Equilibrium guidelines for the evaluation of hearing preservation in acoustic neuroma (vestibular schwannoma). American Academy of Otolaryngology–Head and Neck Surgery. Otolaryngol Head Neck Surg. 1995;113:179–180. 7675475 10.1016/S0194-5998(95)70101-X

[12] Welling DB Glasscock ME III Woods CI Jackson CG. Acoustic neuroma: a cost-effective approach. Otolarynogology Head Neck Surg. 1990;103:364–370. 10.1177/019459989010300305 2122364

[13] Margolis RH Saly GL. Asymmetric hearing loss: definition, validation, and prevalence. Otol Neurotol. 2008;29:422–431. 18418281 10.1097/MAO.0b013e31816c7c09

[14] Urben SL Benninger MS Gibbens ND. Asymmetric sensorineural hearing loss in a community-based population. Otolaryngol Head Neck Surg. 1999;120:809–814. 10352431 10.1016/S0194-5998(99)70318-9

[15] Schlauch RL. Evaluating hearing threshold differences between ears as a screen for acoustic neuroma. J Speech Hear Res. 1995;38:1168–1175. 8558885 10.1044/jshr.3805.1168

[16] Dawes PJD Jeannon JP. Audit of regional screening guidelines for vestibular schwannoma. J Laryngol Otol. 1998;112:860–864. 9876377 10.1017/s0022215100141891

[17] Cueva RA. Auditory brainstem response versus magnetic resonance imaging for the evaluation of asymmetric sensorineural hearing loss. Laryngoscope. 2004;114:1686–1692. 15454755 10.1097/00005537-200410000-00003

[18] Sheppard IJ Milford CA Anslow P. MRI in the detection of acoustic neuromas: a suggested protocol for screening. Clin Otolaryngol. 1996;21:301–304. 8889293 10.1111/j.1365-2273.1996.tb01074.x

[19] Hunter LL Ries DT Schlauch RS Levine SC Ward WD. Safety and clinical performance of acoustic reflex tests. Ear Hear. 1999;20:506–514. 10613388 10.1097/00003446-199912000-00006

[20] Pena I Chew EY Landau BP Breen JT Zevallos JP Vrabec JT. Diagnostic criteria for detection of vestibular schwannomas in the VA population. Otol Neurotol. 2016;37:1510–1515. 27755456 10.1097/MAO.0000000000001251

[21] Mirza B Wang W Wang J Choi H Chung NC Ping P. Machine learning and integrative analysis of biomedical big data. Genes (Basel). 2019;10: 87. 30696086 10.3390/genes10020087 PMC6410075

[22] Carey GE Jacobson CE Warburton AN. Machine learning for vestibular schwannoma diagnosis using audiometrie data alone. Otol Neurotol. 2022;43:e530–e534. 35617004 10.1097/MAO.0000000000003539

[23] Lysaght A Kao S Paulo J Merchant S Steen H. The proteome of human perilymph. NIH Public Access Author Manuscr. 2011;10:3845–3851. 10.1021/pr200346q PMC3179892 21740021

[24] Zhang Y Long J Ren J Huang X Zhong P Wang B. Potential molecular biomarkers of vestibular schwannoma growth: progress and prospects. Front Oncol. 2021;11:1–14. 10.3389/fonc.2021.731441 PMC8503266 34646772

[25] Lassaletta L Calvino M Morales-Puebla JM. Biomarkers in vestibular schwannoma–associated hearing loss. Front Neurol. 2019;10:1–7. 31620068 10.3389/fneur.2019.00978 PMC6759574

[26] Hannan CJ Lewis D O’Leary C. The inflammatory microenvironment in vestibular schwannoma. Neuro-Oncology Adv. 2020;2:1–12. 10.1093/noajnl/vdaa023 PMC7212860 32642684

