# Supplementary material for: MRI Screening in Vestibular Schwannoma: A Deep Learning-based Analysis of Clinical and Audiometric Data
Source: Otol Neurotol Open. 2023 Mar 9;3(1):e028. doi: 10.1097/ONO.0000000000000028 (PMC10950172; doi:10.1097/ONO.0000000000000028)
Supplement: Supplementary file 1 [file ono-3-e028-s001.pdf]

Supplemental Figures

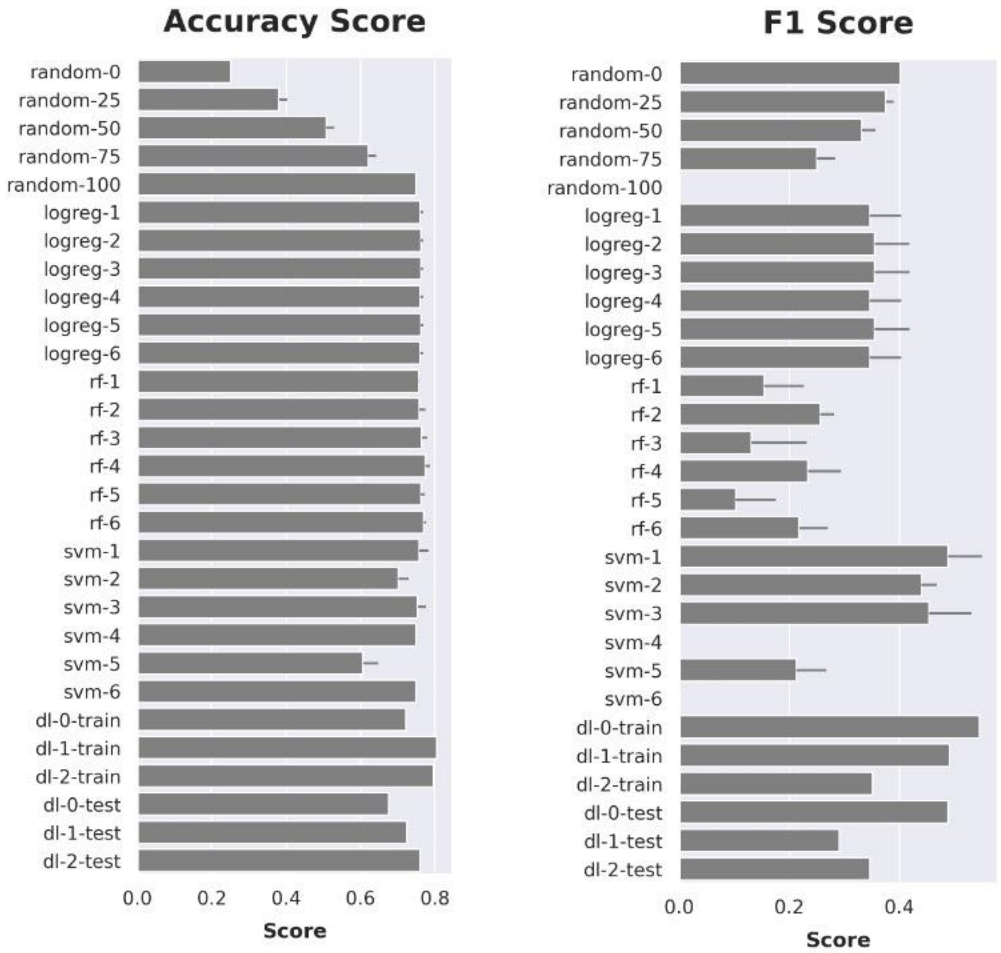

Supplemental Figure 1: Assessment of Machine-learning and Deep-learning-based classification performance by accuracy and F1 score metrics. A, Accuracy of machine learning-based classification models including logistic regression (logreg-1-6), random forest (rf-1-6), support vector machines (svm-1-6), and deep learning-based classification models including deep learning training (dl-0-2-train) and test (dl-0-2-test) datasets are compared against random (random-0-100) where a percentage (0-100) of ears are designated as having vestibular schwannoma (VS). Accuracy is defined as the ratio of correctly predicted cases (positive or negative) divided by all cases. **B,** F1 score of machine learning- and deep learning-based classification models. The F1 score is the weighted average of precision and recall, taking into account both false positives and false negatives into account and is useful in situations where uneven class distribution exists.
